# LARP6 suppresses colorectal cancer progression through *ZNF267/SGMS2*-mediated imbalance of sphingomyelin synthesis

**DOI:** 10.1186/s13046-023-02605-4

**Published:** 2023-01-24

**Authors:** Xiaoli Long, Xunhua Liu, Ting Deng, Jianxiong Chen, Jiawen Lan, Sijing Zhang, Miao Zhou, Dan Guo, Jun Zhou

**Affiliations:** 1 Department of Pathology, Nanfang Hospital, Southern Medical University, Guangzhou, 510515 China; 2 Department of Pathology, School of Basic Medical Sciences, Southern Medical University, Guangzhou, 510515 China; 3 Department of Pathology, YunFu People’s Hospital, Yunfu, 527300 China; 4 Department of Pharmacy, Nanfang Hospital, Southern Medical University, Guangzhou, 510515 China

**Keywords:** *LARP6*, *ZNF267*, *SGMS2*, Metastasis, Sphingolipid, Autophagy

## Abstract

**Background:**

With increasing incidence and mortality, colorectal cancer (CRC) seriously endangers human health. *LARP6*, a member of La-related protein (*LARP*) family, is a RNA binding protein and probably associates with CRC progression, but its specific roles and mechanisms in CRC still remain unknown.

**Method:**

Quantitative real-time PCR (qPCR), western blot, and immunohistochemistry were employed to examine LARP6 expression in CRC tissues. Using the stable *LARP6* overexpression or interference CRC cell lines, the effect of LARP6 on CRC progression were evaluated. High-throughput RNA immunoprecipitation sequencing (RIP-seq) and a series of relevant experiments were conducted to explain how LARP6 functions. SPSS software was used for statistical analysis.

**Result:**

In this study, we found that *LARP6* expression is downregulated in CRC and correlates with patients’ overall survival and relapse-free survival. Furthermore, altered LARP6 expression influences CRC cells invasion and metastasis. Mechanically, we discovered that LARP6 bind *ZNF267* mRNA and regulated its stability and translation. LARP6 inhibited expression of *SGMS2*, a downstream target of ZNF267, resulting in ceramide and sphingomyelin imbalance in CRC cells. Interestingly, LARP6 also enhances autophagy activity of CRC cells, and the effect was at least partially determined by the inhibition of SGMS2-mediated sphingomyelin synthesis.

**Conclusion:**

Our study showed how *LARP6/ZNF267/SGMS2* axis influence CRC progression, which contributes to further understanding of the molecular mechanisms underlying CRC development.

**Supplementary Information:**

The online version contains supplementary material available at 10.1186/s13046-023-02605-4.

## Background

Colorectal cancer (CRC) is one of the most common malignant tumors worldwide with increasing incidence and mortality [1]. Although targeted therapy and immunotherapy have greatly improved the treatment of CRC in recent years, recurrence and metastasis remain the leading cause of cancer-related death. Therefore, it is crucial to elucidate the molecular mechanisms underlying CRC progression and metastasis. Interestingly, in our unpublished work with differential gene expression analysis, we noticed that *LARP6* expression is gradually decreased in matched adjacent normal tissues, CRC tissues without distant metastasis and with distant metastasis, suggesting that *LARP6* may be related to the occurrence and development of CRC.

La ribonucleoprotein domain family member 6 (*LARP6*), also known as Acheron, belongs to the La-related protein (*LARP*) family, which includes the following members: *LARP1*, *LARP1b*, *SSB*, *LARP4*, *LARP4b*, *LARP6* and *LARP7*. With a highly conserved La Module, all members of this family possess characteristics and functions of RBPs [2–4]. Type I collagen mRNA is the first reported LARP6-combined RNA target [5, 6], and although many other RNA targets have been identified [7], the role of LARP6 as RBP still deserves further exploration. Furthermore, LARP6 is also involved in some tumor-related cellular processes, such as cell survival, angiogenesis, motility and the like [7–14]. More importantly, it has been reported that multiple members of LARP family are related to occurrence and development of cancer [2, 15–18], but it is less well studied in CRC. Our prognosis analysis indicated that *LARP6*, but not other *LARP* members, significantly relates to CRC prognosis in different GEO datasets (Fig. S1A-N) [see Additional file 2]. Specifically, *LARP6* is up-regulated in basal-like breast cancer and promotes its progression [18]. In addition, expression dysregulation of *LARP6* has also been found in lung cancer and gastric cancer [19, 20], but its relationship with CRC still remains unknown.

In this study, we identified *LARP6* as a suppressor of CRC metastasis. Mechanically, *LARP6* induce ceramide and sphingomyelin imbalance and increase autophagy activity by regulating *ZNF267/SGMS2* axis in CRC cells. In conclusion, our study characterized how LARP6/ZNF267/SGMS2 axis functions in CRC metastasis, and provided a potential therapeutic target for CRC treatment.

## Materials and methods

### Cell culture

Normal human colon mucosal epithelial cell (NCM460) and human CRC cell lines (SW480, DLD1, RKO, LOVO, CACO2, HCT116, and SW620) were obtained from the cell bank at the Chinese Academy of Sciences (Shanghai, China). Cells were authenticated by short tandem repeat (STR) profiling after receipt and were propagated for less than 6 months after resuscitation. Maintained in a humidified chamber containing 5% CO2 at 37 °C, cells were routinely cultured in RPMI 1640 supplemented with 10% fetal bovine serum (FBS; Gibco, USA) and 1% antibiotics (Gibco, USA).

### Patient samples

The Institute Research Medical Ethics Committee of Nanfang Hospital (Guangzhou, China) granted approval for this study. Informed consent was obtained from all patients. 61 pairs of fresh CRC tissues and matched adjacent normal tissues were collected randomly from primary CRC patients without any treatment before surgery in Nanfang Hospital from 2018 to 2020, and 49 pairs of them were analyzed by RT-PCR, and 12 pairs were analyzed by WB. Paraffin-embedded CRC tissues and matched adjacent normal tissues of 165 primary CRC patients were gathered in Nanfang Hospital from 2017 to 2021, and none of the patients received any preoperative chemotherapy or radiotherapy before surgery. Serial sections of paraffin-embedded CRC tissues used for IHC were obtained from the above 165 patients. Three cohorts of tissue samples for RT-PCR, WB and IHC are obtained from different primary CRC patients. The details of clinical information is provided in the Additional files [see Additional file 1].

### Mice

BALB/c nude mice (male, 4 weeks old) were provided by the animal center of Guangdong Province. All mice experiments were approved by the Committee on the Ethics of Animal Experiments of Southern Medical University. All animal studies were strictly complied with regulations in the Guide for the Care and Use of Laboratory Animals of the National Institutes of Health.

### RNA extraction and quantitative real-time PCR (qPCR, RT-PCR)

According to the manufacturer’s instruction, total RNA in cells or tissues were extracted with TRIzol reagent (TaKaRa, Japan). cDNAs were generated with PrimeScript RT-PCR Kit (TaKaRa, Japan). RT-PCR was conducted using SYBR Premix Ex Taq (TaKaRa, Japan) on ABI7500 Real-time PCR system (Applied Biosystems, Foster City, USA). Relative mRNA expression was calculated according to the work by Pfaffl [21].

### Protein extraction and western blotting (WB)

With RIPA buffer containing a protease inhibitor and a phosphatase inhibitor (FDbio, China), cells and tissues were lysed, then proteins were harvested. Proteins were separated by SDS-polyacrylamide gel electrophoresis (PAGE) and electrotransferred onto a polyvinylidene difluoride (PVDF) membrane (Merck millipore, USA). Having been incubated with primary and secondary antibodies, protein expression was visualized with an enhanced chemiluminescence system. Primary antibodies used are listed as follows: anti-LARP6 antibody (1:500; Abcam, Britain), anti-GAPDH antibody (1:1000; Proteintech, USA), anti-ZNF267 antibody (1:1000; Novus, USA), anti-SGMS2 antibody (1:1000; Novus, USA), anti-P62 antibody (1:1000; Proteintech, USA), anti-LC3B antibody (1:500; ABclonal, USA).

### Immunohistochemistry (IHC)

IHC staining of paraffin-embedded human or mice tissues was carried out according to protocols. With dewaxing, hydration, antigen repair and block successively, sections were incubated in primary antibodies overnight at 4 °C. Next day, sections were put in room temperature for 1 h to rewarm, followed by secondary antibody incubation for 1 h in room temperature. Having been stained with 3,3-diaminobenzidine (DAB, ZSGB-BIO, China), the slides were counterstained with hematoxylin, dehydrated and mounted. Primary antibodies used are listed below: anti-LARP6 antibody (1:50; Abcam, Britain), anti-ZNF267 (1:100; Novus, USA), anti-SGMS2 (1:300; Novus, USA).

Performed by two independent pathologists blinded to the clinical data, IHC staining was scored as the product of staining intensity (0–3) and percentage of positive area (0–100) (overall score range = 0–300).

### Lentivirus and plasmids transfection

Lentiviral vectors were constructed by GENECHEM Biotech at Shanghai, China (http://genechem.bioon.com.cn/). Luciferase-tagged LARP6-overexpressed vectors and control vectors were transfected into SW480 and DLD1 cells, while LARP6 shRNA and control short hairpin RNA were transfected into CACO2 and SW620 cells to generate cells with stable knockdown of LARP6. Transfection was carried out in accordance with protocol. Briefly, cells (1 × 10^5^ per well) grown in 6-well plates were transducted with lentivirus for 24 h, followed by selection with puromycin (4 μg/ml) for 48 h post transduction for 4–5 days. Effect of overexpression or knockdown was analyzed by qRT-PCR and western blotting.

Overexpression and knockdown of *ZNF267* and *SGMS2* in CRC cells were achieved by plasmids and siRNA (small interfering RNA) transfection using Lipofectamine 3000 (Life Technologies, USA). Transfection protocol was resemble to Lentivirus.

### CCK8 and colony formation assay

For cell proliferation assay, cells (1 × 10^3^ cells per well) were seeded on 96-well plates and cell proliferation were determined for 7 days with cell counting kit-8 (DOJINDO Laboratories, Japan) according to instructions. For colony formation assay, cells (500 cells per well) were seeded on 6-well plates and cultured for 2 weeks. Colonies were fixed in methanol and stained with 1% Giemsa. Colonies containing more than 50 cells for each well were counted. All observations were reproduced at least three times in independent experiments.

### Migration and invasion assay

For cell migration analysis, 2 × 10^5^ cells suspended in serum-free media were seeded into the 8-μm-pore upper chambers and incubated in RPMI1640 with 10% FBS of the lower chamber of 24-well plates (Corning, USA). After regular culture for 20–48 h, cells were fixed with methanol and then stained with Giemsa. Migratory cells were calculated in five randomly chosen fields (magnification, 200×). For invasion assay, the upper chamber was coated with 200 μg/ml Matrigel (Corning, USA) and 2 × 10^5^ cells were seeded into the upper chamber. Other procedures were resemble to migration assay. All experiments were repeated at least three times.

### Orthotopic colorectal cancer mice model

Luciferase-tagged LARP6 shRNA and control shRNA were transfected into CACO2 cells. 2 × 10^6^ CACO2 cells with or without LARP6 knockdown were then injected into the subserous layer of the cecum in nude mice (BALB/c-nu/nu, male, 4 weeks old, 8 mice per group). To assess metastasis of CRC in mice model, at 60 days after surgery, mice were injected intraperitoneally with luciferase substrate (Promega), and luciferase activity using an instrument (FX Pro, USA) was non-invasively detected. The mice were then killed, with intestine and liver tissues separated for further assays.

### RNA immunoprecipitation (RIP)

Cells were gathered for RNA immunoprecipitation assay as kit instructed (Abcam, Britain). Cell lysate was incubated in anti-LARP6 antibody overnight at 4 °C, followed by conjugating to protein dynabeads, with serum (IgG) as a control group. RNA was extracted using TRIzol following manufacturer’s instructions (TaKaRa, Japan). cDNAs generation and RT-qPCR were performed as described earlier. The fold enrichment for each target was measured by comparing the Ct values of LARP6-immunoprecipitated fraction to the IgG isotype fraction and normalized using the ΔCt formula. GAPDH was used as a negative control.

### Biotinylated RNA pull-down assay

Biotin-labeled ZNF267 RNA probe was designed, synthesized and purchased from Sangoon Biotech (Shanghai, China). With continuous rotation, purified biotinylated *ZNF267* RNA probe was incubated with total cell lysates for 1 h at room temperature. Complexes were isolated with streptavidin-conjugated Dynabeads (Invitrogen, USA), followed by boiling with SDS-PAGE loading buffer for 5 min. The pull-down materials were subsequently analyzed by western blotting with LARP6-specific antibody.

### mRNA stability analysis

Cells were treated with actinomycin D (2 μg/ml; FDbio, China) for 0, 0.5, 1, 3, 5 and 7 h, followed by RNA extraction at every time points. cDNA generation and RT-PCR were the same as described earlier. *ZNF267* mRNA abundance was analyzed, with *GAPDH* as the endogenous control. *ZNF267* mRNA level was normalized to the 0 h time point.

### Polysome analysis

On being equally plated on cell culture dish at the concentration of approximately 20–25%, cells were maintained for 2–3 days until became 90% confluent. As it was mentioned [22], cells were treated with 100 μg/ml cycloheximide (CHX, Selleckchem, China) for 10 min, prior to lysing in 300 μL of lysis buffer. Nuclei and membrane debris were then removed by centrifuging at 12000 g, 10 min. The lysate was loaded onto a sucrose gradient (10–50% sucrose(w/v)) and centrifuged in a SW41Ti rotor (Beckman, USA) for 1.5 h at 39000 rpm at 4 °C. Fractions were collected by density gradient fractionation system Piston Gradient Fractionator™ (BIOCOMP, Canada). cDNA generation and RT-PCR were the same as described earlier.

### Chromatin immunoprecipitation (ChIP)

ChIP was carried out as Chromatin Immunoprecipitation kit (Abcam, Britain) described. Immunoprecipitation reactions were performed with anti-ZNF267 antibody (5 μg; Novus, USA), and IgG was as a control. Purified DNA was used for RT-PCR, and primers were designed specific to *SGMS2* promoter. The fold enrichment was measured by comparing the Ct values of ZNF267-immunoprecipitated fraction to the IgG isotype fraction and normalized using the ΔCt formula.

### Dual-luciferase reporter gene assay

All experiments were performed according to the kit instructions (Yeasen, China). Cells were inoculated in a 96-well plate and cell lysis buffer was transferred to the black microplate, then firefly luciferase reaction solution was added and the firefly luciferase activity was determined. After inocubating with Renilla luciferase reaction solution, the activity was measured.

### Total ceramide and sphingomyelin level detection

With quantitative cell or tissue lysate collected, the total ceramide level was measured using a human ceramide ELISA Kit (Enzyme-linked Biotechnology, Shanghai, China) according to the manufacturer’s instructions, and the total sphingomyelin level was measured using a human sphingomyelin Kit (Abcam, Britain).

### Transmission electron microscopy (TEM)

With cell pellets about the size of a grain of rice collected, sample was fixed with 2% glutaraldehyde at 4 °C overnight. Samples were then postfixed with 1% OsO4 dissolved in 0.1 M PBS for 2 h and dehydrated using an ascending gradual series (50–100%) of ethanol and infiltrated with propylene oxide. After sectioning and staining with uranyl acetate and lead citrate, samples were viewed via TEM (HITACHI, HT7700, Japan).

### Autophagy flux detection

Transfected with RFP-GFP-LC3B lentivirus, cells with LARP6 overexpression or interference and control cells were fixed using 4% paraformaldehyde and counterstained with DAPI. With confocal microscope, autophagy activity was assessed by quantitation of the number of red and yellow puncta in cells, counting at least 10 cells per group.

### Statistical analysis

Each assay was performed in at least three independent experiments. Statistical analysis were finished using SPSS software (version 23.0, IBM Corp, Armonk, NY, USA). A two-tailed, unpaired, or paired Student t test was used to compare the variables of two groups, and one-way or two-way ANOVA were performed for multi-group comparisons. *P* < 0.05 was considered statistically significant (*P < 0.05, **P < 0.01, ***P < 0.001, ns means no statistic difference). The error bars represent Mean ± SD.

## Results

### LARP6 expression is down-regulated in CRC and low expression is associated with poor prognosis

**Fig. 1:**
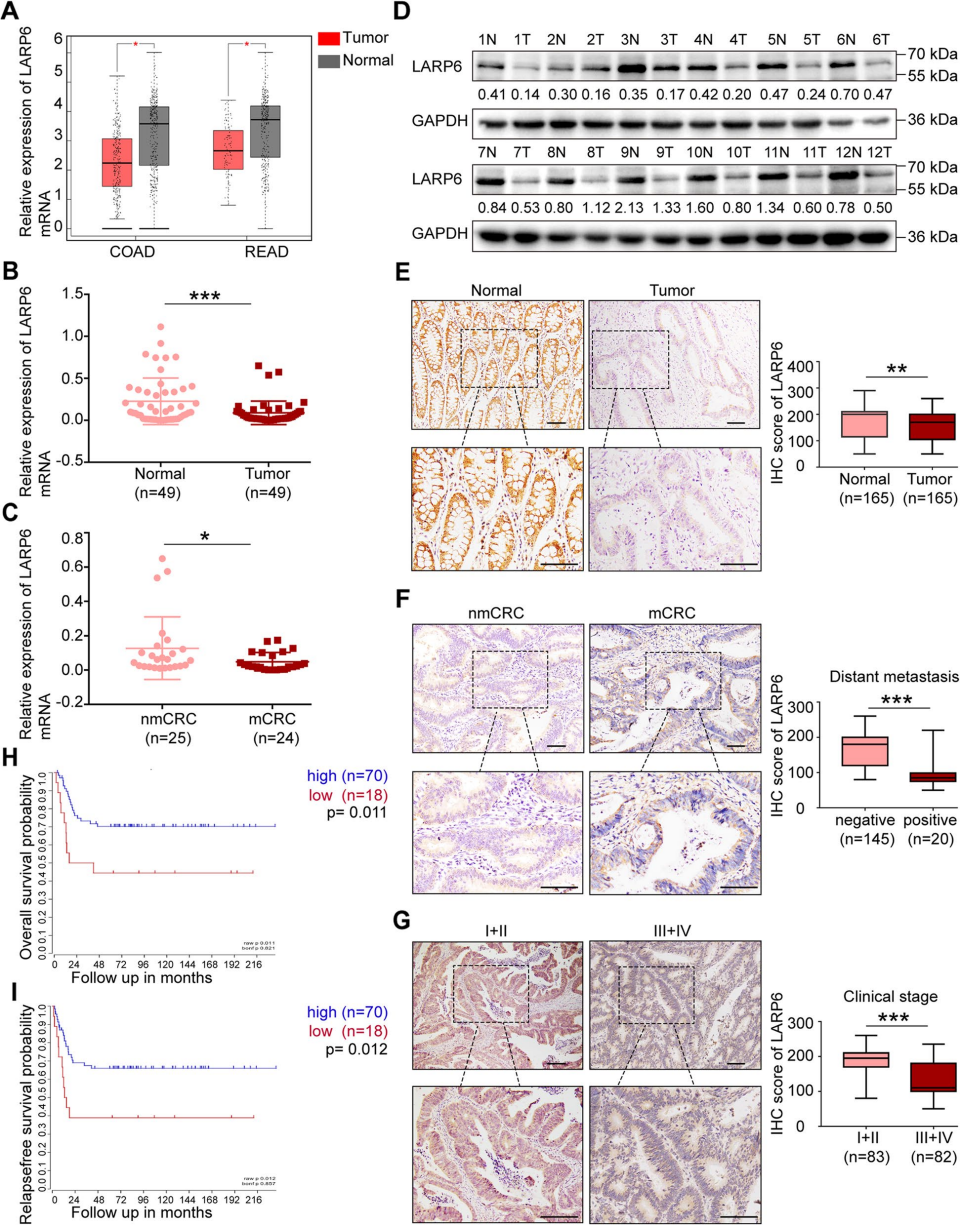
LARP6 expression is down-regulated in CRC and low expression is associated with poor prognosis. **A** Online analysis of LARP6 mRNA expression in CRC samples from Gene Expression Profiling Interactive Analysis (GEPIA). [COAD]: colon adenocarcinoma, [READ]: rectal adenocarcinoma. The red * means *P* < 0.01. **B** qPCR results of LARP6 mRNA expression in 49 paired fresh CRC tissues and matched adjacent normal tissues. **C** LARP6 mRNA expression in CRC tissues with or without lymph node metastasis was analyzed by qPCR. [mCRC]: CRC with lymph node metastasis; [nmCRC]: CRC without lymph node metastasis. **D** LARP6 protein expression through WB assay in 12 paired fresh CRC tissues and matched adjacent normal tissues. [T]: CRC tissues, [N]: matched adjacent normal colon tissues. **E** Representative IHC staining images of LARP6 in 165 paraffin-embedded primary CRC tissues and matched adjacent normal tissues. Statistical results are shown in right panels. **F** Representative IHC staining images of LARP6 in CRC tissues with distant metastasis and without. Statistical results are shown in right panels. **G** Representative IHC staining images of LARP6 in stage I + II and III + IV CRC tissues. Statistical results are shown in right panels. **H-I** Online Kaplan–Meier survival analysis of overall survival rate (**H**) and relapse-free survival rate (**I**) between high LARP6 expression group (blue line) and low LARP6 expression group (red line). *P < 0.05, **P < 0.01, ***P < 0.001, ns means no statistic difference. The error bars represent Mean ± SD

To explore whether *LARP6* is involved in CRC development, we firstly analyzed *LARP6* expression profile on GEPIA and discovered a lower *LARP6* expression in CRC compared with normal mucosa (Fig. 1A). Next, a CRC cohort containing 61 pairs of fresh CRC tissues and matched adjacent normal tissues were collected from untreated primary CRC patients. In this cohort, 49 paires of samples were analyzed by qPCR and 12 paires of tissues were analyzed by WB. Consistent with the online findings, our qPCR and WB results also indicated that *LARP6* expression is lower in CRC tissues than in normal mucosa (Fig. 1B-D). Interestingly, we noted that *LARP6* expression is significantly reduced in CRC tissues of primary CRC patients with lymph node metastasis compared with those without lymph node metastasis (Fig. 1C). IHC staining of 165 paraffin-embedded primary CRC tissues and matched adjacent normal tissues gathered from Nanfang Hospital from 2017 to 2021 showed that LARP6 expression was down-regulated in CRC (Fig. 1E). In order to clarify the clinical significance of *LARP6* dowen-regulated expression, we comprehensively analyzed clinical characteristics and LARP6 expression in IHC CRC cohort, and the results suggested that LARP6 expression is negatively correlated with T-stage, lymph node status, distant metastasis and clinical stage (Fig. 1F-G, Table S1) [see Additional file 2], but not with age, gender and tumor size (Table S1) [see Additional file 2]. Besides, online survival analysis indicated that CRC patients with low *LARP6* expression present a poorer overall and relapse-free survival (Fig. 1H-I, Fig. S1A-N) [see Additional file 2]. Taken together, these results suggested that *LARP6* is lowly expressed in CRC and the down-regulation of *LARP6* may be involved in CRC progression.

### LARP6 inhibits CRC cell invasion and metastasis in vitro and in vivo

**Fig. 2:**
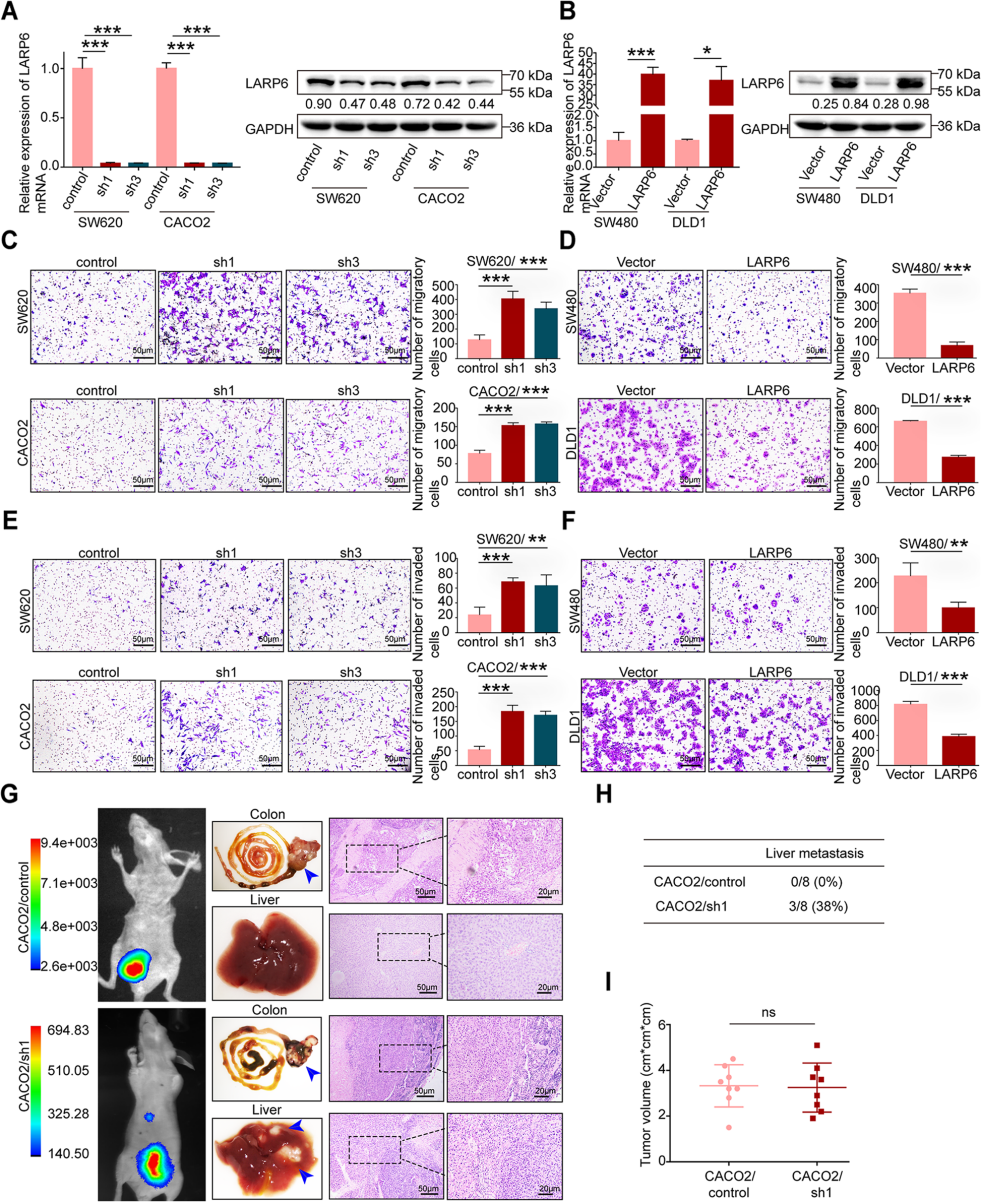
LARP6 inhibits CRC cell invasion and metastasis in vitro and in vivo. **A-B** Identification of LARP6 overexpression (**B**) and interference (**A**) effects through qPCR and WB. **C-D** Transwell migration assays in CRC cells with LARP6-overexpression (**D**) or knockdown (**C**). Statistical results are shown in right panels (*N* = 3). **E-F** Marigel transwell invasion assays of LARP6-overexpression (**F**) or LARP6-knockdown (**E**) in CRC cells. Statistical results are shown in right panels (*N* = 3). **G** Representative images of orthotopic CRC mice model constructed with LARP6-interfered CACO2 cells or control cells: fluorescence in vivo imaging at 60 days after injection (left), macroscopic pictures of colon and liver samples (middle), and H&E staining of liver tissues and primary colon tumor tissues (right). The blue arrow indicates the tumor. **H** Summary results of the nude mice presenting with liver metastasis between LARP6-knockdown group and control group. **I** The primary tumor growth was measured by volume (cm^3^). *P < 0.05, **P < 0.01, ***P < 0.001, ns means no statistic difference. The error bars represent Mean ± SD

To evaluate how LARP6 influences biological behavior of CRC cell, we firstly detected endogenous *LARP6* expression in 7 CRC cells (Fig. S2A-B) [see Additional file 2]. SW480 and DLD1, two cell lines with low *LARP6* expression, were selected to construct stable *LARP6* overexpression cell lines (Fig. 2B), while SW620 and CACO2 were chosen for stable interference cell lines (Fig. 2A). CCK8 and clone formation assays showed that LARP6 has no significant effect on the proliferation of CRC cells in vitro (Fig. S2C-D) [see Additional file 2]. However, as detected by transwell assays, *LARP6* overexpression markedly weakened the migration and invasion ability of SW480 and DLD1 cells (Fig. 2D, F), while *LARP6* knockdown exerted an opposite effect (Fig. 2C, E).

Next, we wondered whether LARP6 also affects CRC progression in vivo. CACO2 cells, transfected with luciferase-tagged *LARP6* shRNA and control shRNA separately, were injected into the caecum to establish an orthotopic CRC mice model (8 mice per group). On the 60th day after injection, mice were injected intraperitoneally with luciferase substrate, and the luciferase signals, reflecting of the location and the size of tumors, were detected using a multimodel animal imaging system. Mice in *LARP6* interference group presented more fluorescent signals in liver than the control group (Fig. 2G), but there was no significant difference in volume of primary tumor between two groups (Fig. 2I). As confirmed by H&E staining of tissues (Fig. 2G-H), *LARP6* interference group exhibited a higher liver metastasis rate than the control group. This indicated that the *LARP6* interference group had a stronger liver metastasis ability. Together, these results showed that LARP6 inhibits liver metastasis of CRC in vivo.

### LARP6 up-regulates ZNF267 expression by binding and stablizing ZNF267 mRNA

**Fig. 3:**
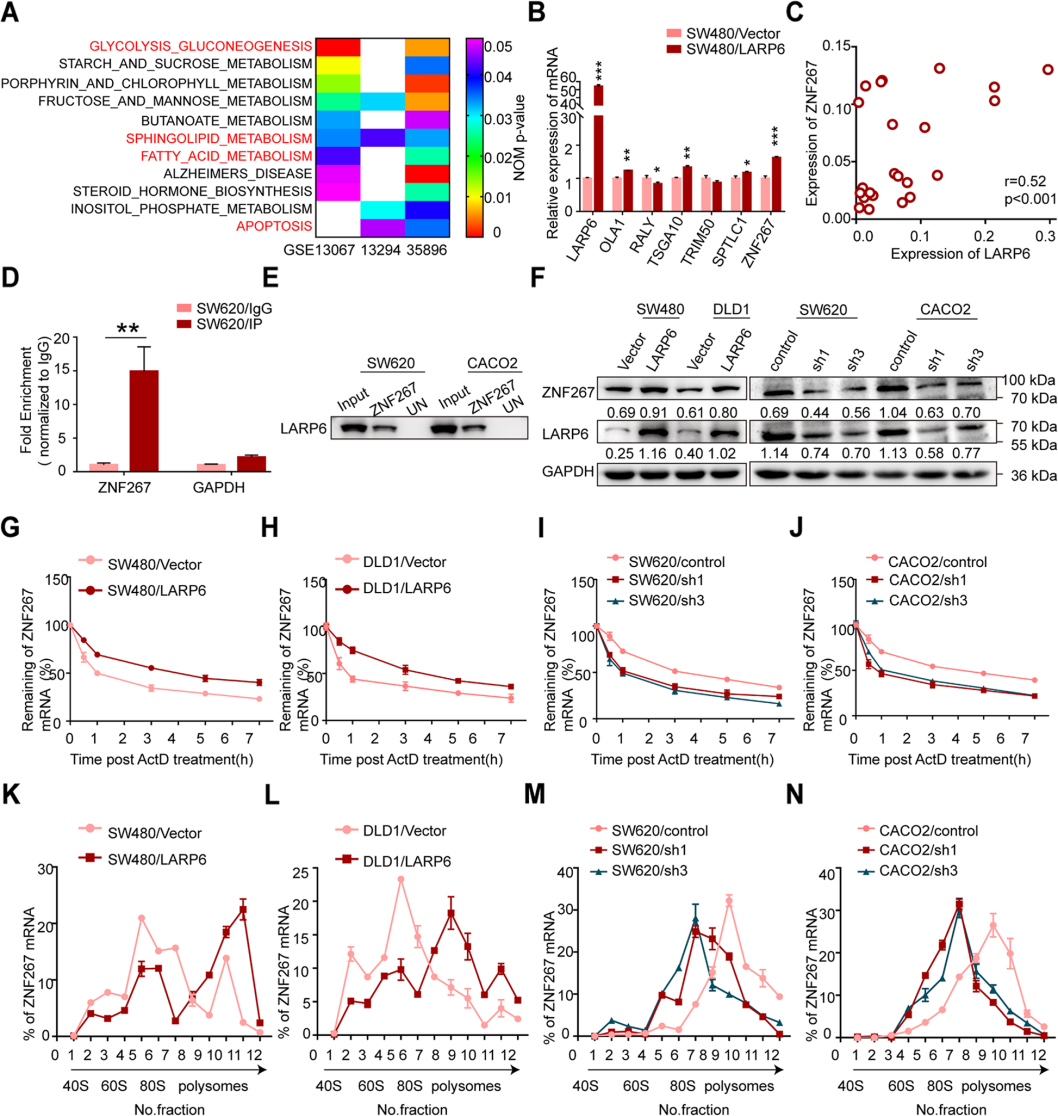
LARP6 up-regulates ZNF267 expression by binding and stablizing ZNF267 mRNA. **A** KEGG enrichment analysis of LARP6 using three different GEO datasets. **B** qPCR analysis of candidate genes in LARP6-overexpressed SW480 cells and control cells. **C** Correlation analysis of mRNA expression between LARP6 and ZNF267 in 49 paired fresh CRC tissues and matched adjacent normal tissues. **D** RIP-qPCR assay with anti-LARP6 antibody in SW620 cells (*N* = 3). GAPDH as a negative control. **E** RNA-pull down assay with ZNF267 RNA probe in CRC cells. Input represents 1% of lysate used in pulldown reactions. UN indicates a control pulldown containing beads only. **F** Affection of LARP6 on ZNF267 protein expression was analyzed by WB. **G-J** With a transcription inhibitor ActD, ZNF267 mRNA stability in CRC cells with LARP6 over-expression (**G**-**H**) or knockdown (**I**-**J**) was detected. **K-N** Sucrose gradient fractionation was conducted to analyze the influence of LARP6 on translation activity of ZNF267 mRNA. *P < 0.05, **P < 0.01, ***P < 0.001, ns means no statistic difference. The error bars represent Mean ± SD

To elucidate the molecular mechanism by which LARP6 inhibits CRC metastasis, KEGG enrichment analysis with different GEO datasets was performed. As shown in Fig. 3A, some cancer-related processes, such as apoptosis, oxidative phosphorylation, fatty acid metabolism and sphingolipid metabolism, were significantly enriched in the lower LARP6 expression group.

Considering the fact that transcriptome imbalance of downstream is an important way for RBPs to function in tumors [23–28], we next selected SW620 cells with high endogenous LARP6 expression for high-throughput RNA immunoprecipitation sequencing (RIP-seq) [see Additional file 3]. Interestingly, peak genes in RIP-seq list were significantly associated with phagosome, cell adhesion molecules (CAMs), adherens junction and regulation of actin cytoskeleton [see Additional file 3], which further supports the role of RNA targets in the function of LARP6 in CRC. We preliminarily selected several genes closely related to tumor metastasis in our RIP-seq list, and studied the influence of LARP6 on these mRNA abundance. As shown in our results, mRNA expression of these six genes were affected by *LARP6* overexpression or knockdown in different extent (Fig. 3B, Fig. S2E-G [see Additional file 2]), but *ZNF267* shared a most evident effect. Zinc finger protein 267 (ZNF267), belonging to Kruppel-like zinc finger family, can affect expression of MMP and ADAM protease family members [29–31]. Besides, *ZNF267* is upregulated in HCC and promotes HCC cells proliferation and migration [31]. Though its role in CRC is still unknown, we performed GO analysis using TCGA dataset and found that ZNF267 is related to cell migration, adhesion, cell metabolism and lipid metabolism [see Additional file 4]. These pathways were overlapped with the KEGG enrichment results of LARP6. What’s more, we detected *ZNF267* mRNA expression in 49 pairs of fresh CRC tissues and matched adjacent normal tissues and found that LARP6 is positively correlated with *ZNF267* expression in CRC patients (Fig. 3C).

To further characterize relationship between LARP6 and ZNF267, we firstly performed RIP-qPCR and RNA-pull down experiments, which showed that LARP6 binds to *ZNF267* mRNA (Fig. 3D-E, S2H [see Additional file 2]). Meanwhile, overexpression of LARP6 increased protein level of ZNF267, while LARP6 interference reduced ZNF267 expression (Fig. 3F). RBPs are important regulators of mRNA stability and translation [32–35]. To clarify specific mechanism of above expression regulation, on the one hand, we treated cells with actinomycin D (ActD) and found that ZNF267 transcripts showed a better stability in LARP6 over-expressed cells (Fig. 3G-H), while opposite result was observed in cells with LARP6 knockdown (Fig. 3I-J). On the other hand, *ZNF267* mRNA mainly distributed in polysomes (translationally active ribosome fractions) detected by sucrose gradient fractionation. Specifically, more *ZNF267* mRNA accumulation in polysomes was observed in *LARP6*-overexpressed cells (Fig. 3K-L), while *LARP6* knockdown increased the accumulation of *ZNF267* mRNA in low translational activity portion (Fig. 3M-N). In conclusion, these results suggested that LARP6 binds to *ZNF267* mRNA and increases *ZNF267* expression in a post-transcriptional manner.

### LARP6 constrain CRC invasion through ZNF267

**Fig. 4:**
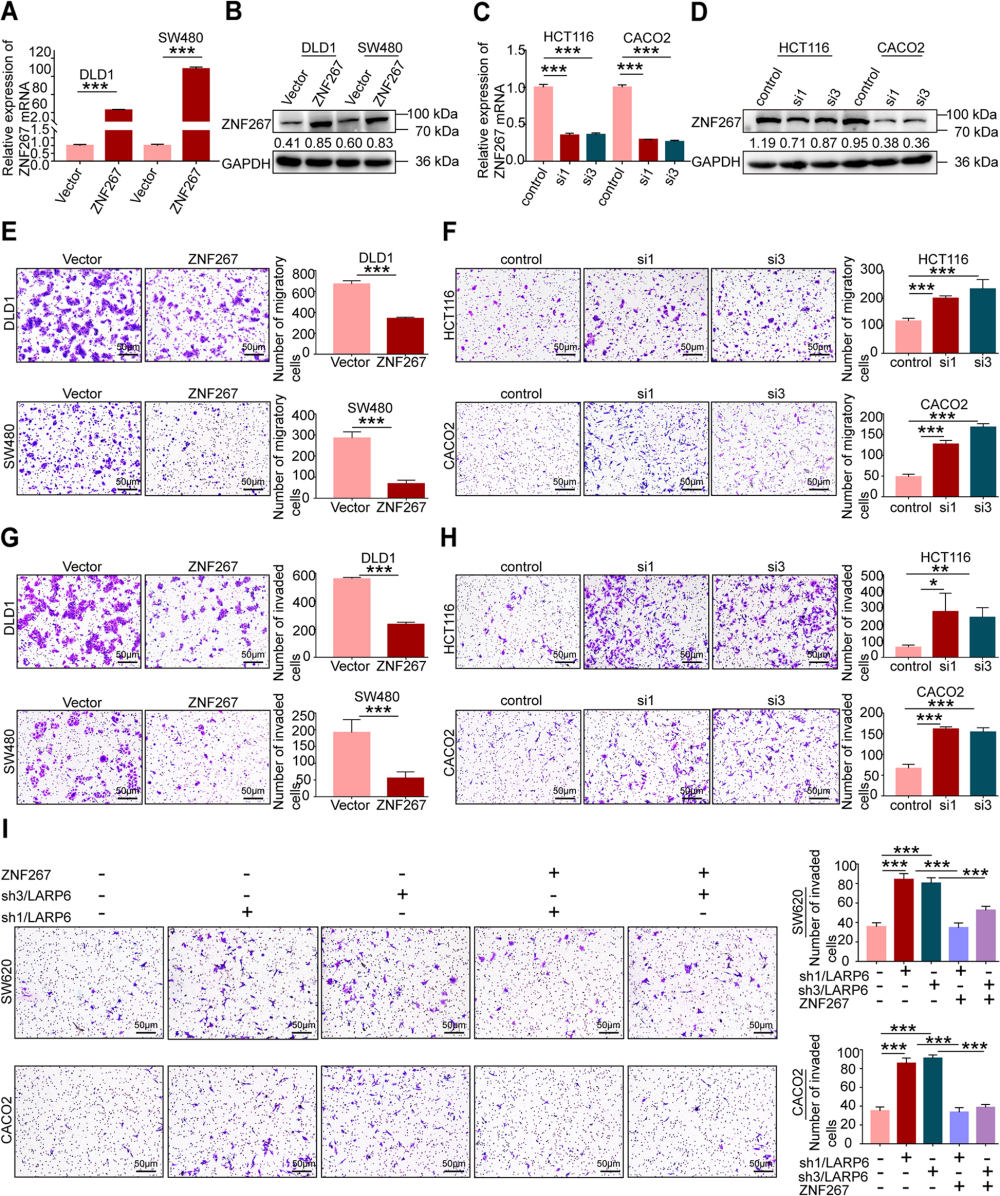
LARP6 constrain CRC invasion through ZNF267. **A-D** qPCR and WB were conducted to identify ZNF267 overexpression (**A**-**B**) and interference (**C**-**D**) effects. **E-F** Transwell migration assays in CRC cells with ZNF267-overexpression (**E**) or knockdown (**F**). Statistical results are shown in right panels (*N* = 3). **G-H** Matrigel transwell experiments illustrated the role of ZNF267 in CRC cell invasion. The right panels show the statistical results (*N* = 3). **I** With forced expression of ZNF267, matrigel transwell assays in LARP6-knockdown cells were conducted. Statistical results are shown in right panels (*N* = 3). *P < 0.05, **P < 0.01, ***P < 0.001, ns means no statistic difference. The error bars represent Mean ± SD

Although studies have suggested that *ZNF267* is involved in tumor progression, its role in CRC metastasis is largely unknown. By constructing *ZNF267* overexpression and knockdown CRC cell lines (Fig. 4A-D, S3A [see Additional file 2]), we performed transwell assays and found that *ZNF267* overexpression inhibited CRC cells migration and invasion (Fig. 4E, G), while *ZNF267* interference obtained opposite results (Fig. 4F, H). These results suggested that ZNF267 may play an vital role in LARP6 constrained CRC invasion and metastasis. Matrigel transwell assay showed that ZNF267 rescued the improved invasion ability that *LARP6* knockdown induced (Fig. 4I, S3B-C [see Additional file 2]). These results showed that ZNF267 can be a target of LARP6 to constrain the invasion of CRC cells.

### By up-regulating ZNF267 expression, LARP6 inhibits SGMS2-mediated sphingomyelin synthesis

**Fig. 5:**
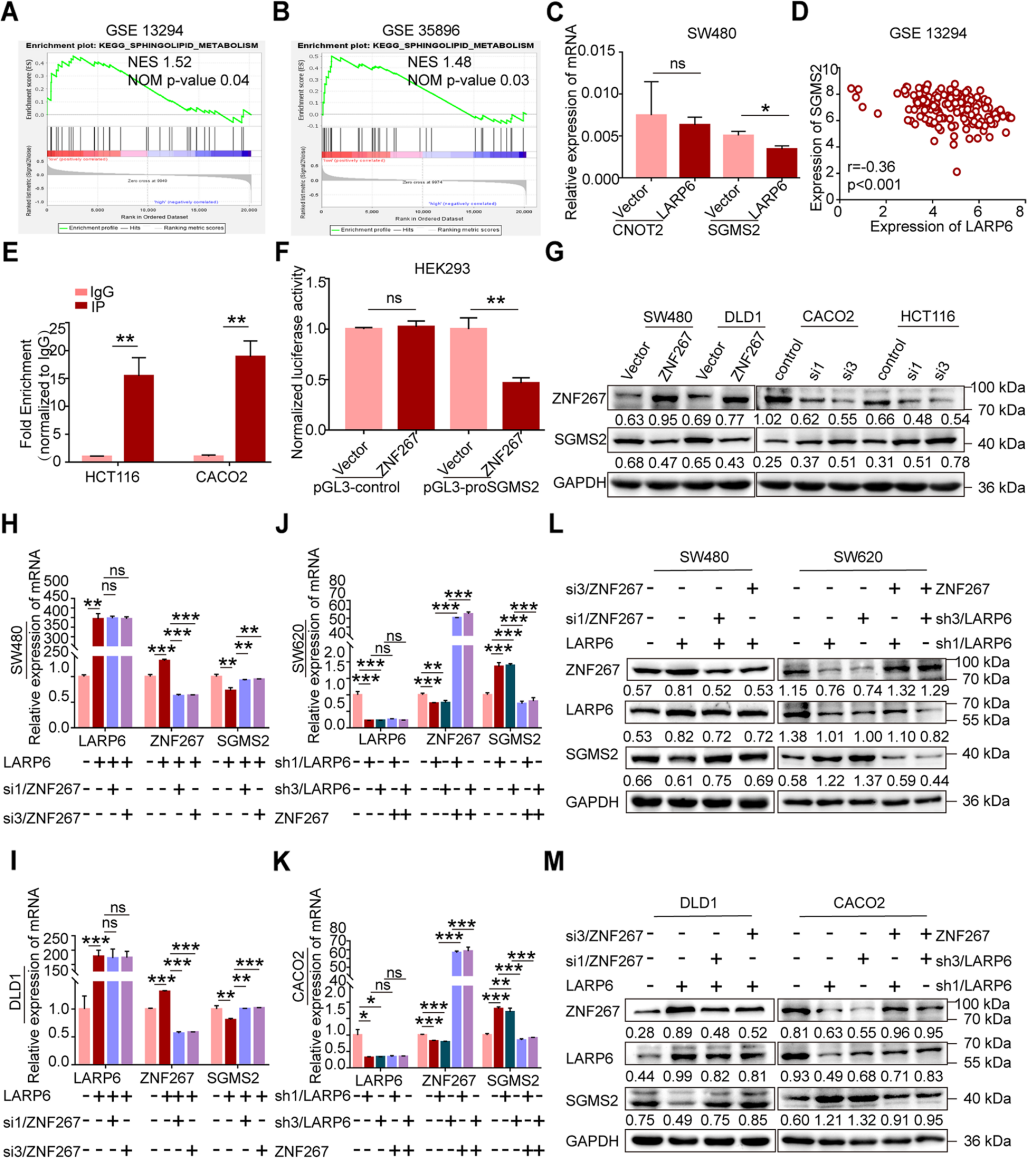
By up-regulating ZNF267 expression, LARP6 suppresses SGMS2 expression. **A**-**B** Relation between LARP6 and sphingolipid metabolism in KEGG enrichment analysis. Sample information were from three different GEO datasets as before. NES: normalized enrichment score. **C** qPCR was used to examine CNOT2 and SGMS2 mRNA expression in SW480 cells upon LARP6 over-expression. **D** Correlation analysis of LARP6 and SGMS2 expression in GEO datasets. r value represents correlation. **E-F** ZNF267 inhibited the transcription of SGMS2: (**E**) CHIP-qPCR and Dual-luciferase reporter gene assay (**F**) verified the transcription inhibition of SGMS2 by ZNF267. **G** The suppression of ZNF267 on SGMS2 protein expression was shown through WB. **H-M** After interference or ectopic expression of ZNF267, qPCR (**H**-**K**) and WB (**L**-**M**) were experimented to detect SGMS2 expression in LARP6 overexpression or knockdown cells. *P < 0.05, **P < 0.01, ***P < 0.001, ns means no statistic difference. The error bars represent Mean ± SD

Next, we sought to investigate how LARP6/ZNF267 axis affects CRC metastasis. As shown in KEGG enrichment analysis (Fig. 3A), LARP6 is highly correlated with oxidative phosphorylation, fatty acid metabolism and sphingolipid metabolism, among which sphingolipid metabolism was significantly enriched in three different datasets and greatly attracted our attention (Fig. 5A-B, S3D [see Additional file 2]). Interestingly, ZNF267 is also closely associated with lipid metabolism in CRC [see Additional file 4], which led us to speculate whether the regulation of LARP6/ZNG267 axis on CRC metastasis relates to lipid metabolism. On the other hand, we tried to mine the downstream of *ZNF267* using CistromeDB (http://cistrome.org/db/#/) (GSM2466511, CistromeDB: 77152) and noticed two genes related to lipid metabolism, *SGMS2* and *CNOT2*. qPCR experiments showed that LARP6 inhibits *SGMS2* mRNA expression but not *CNOT2* (Fig. 5C, S3E [see Additional file 2]). Furthermore, expression correlation analysis using multiple GEO datasets revealed a negative correlation between *LARP6* and *SGMS2* (Fig. 5D, S3F-G [see Additional file 2]), which further supported our hypothesis that SGMS2 may associate with LARP6/ZNF267 axis-mediated CRC progression.

To clarify the regulation of LARP6/ZNF267 axis on *SGMS2* expression, chromatin immunoprecipitation (ChIP) and dual-luciferase reporter gene assays were performed. The results showed that ZNF267 binds to *SGMS2* promoter directly and regulates its transcription (Fig. 5E-F). Meanwhile, negative regulation of ZNF267 on *SGMS2* mRNA and protein expression was also identified (Fig. 5G, S3H-K [see Additional file 2]). Not surprisingly, we also found that LARP6 inhibits *SGMS2* expression in a ZNF267-dependent manner (Fig. 5H-M).

**Fig. 6:**
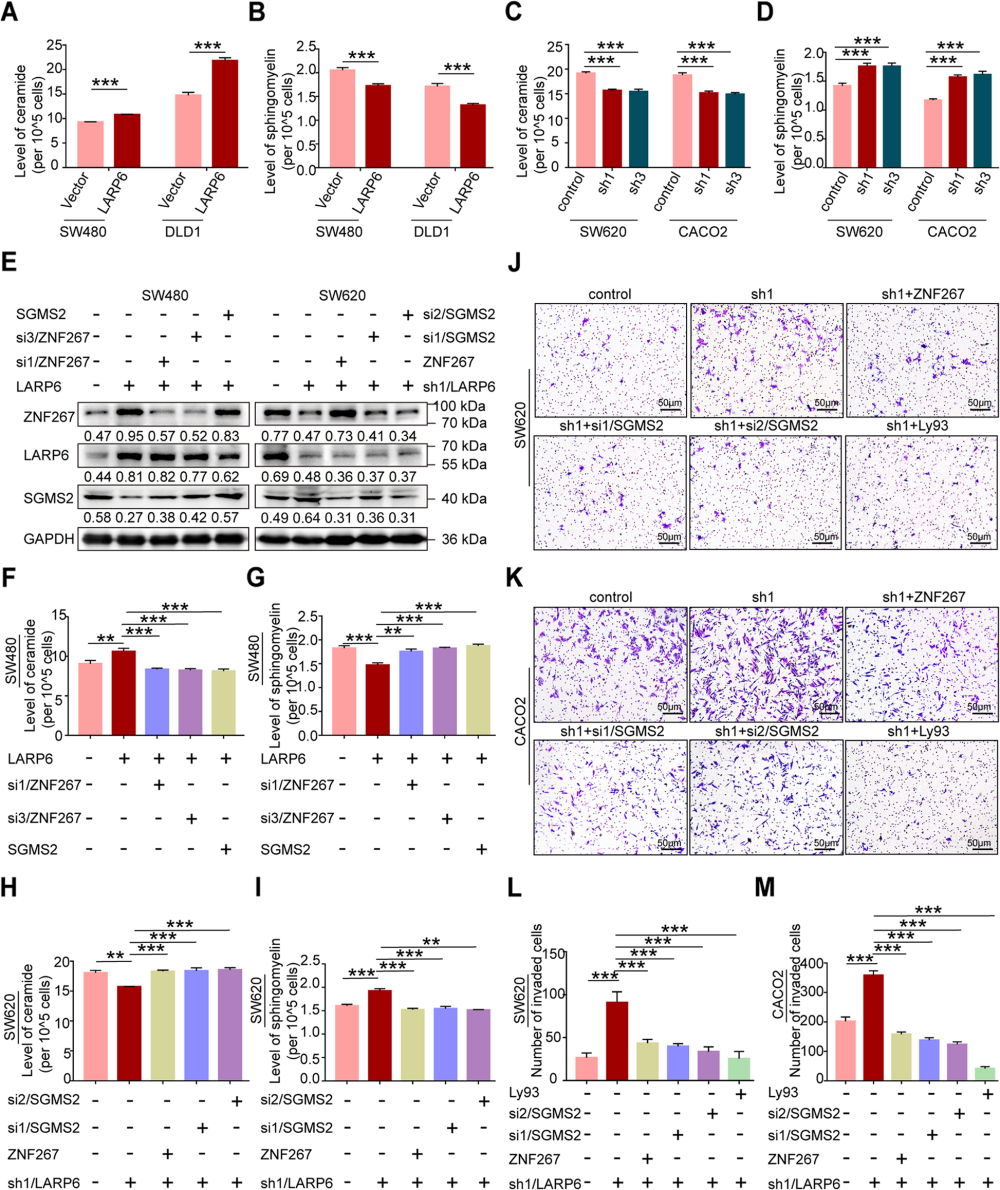
LARP6 inhibits SGMS2-mediated sphingomyelin synthesis. **A-D** Ceramide ELISA Kit and sphingomyelin Kit were used to detect the total ceramide (**A**, **C**) and sphingomyelin (**B**, **D**) level in CRC cells with LARP6 overexpression or interference (*N* = 3). **E-I** In LARP6-overexpressed or interfered CRC cells, total ceramide and sphingomyelin level were detected after forced expression or knockdown of ZNF267 or SGMS2: **E** identification of overexpression or interference of ZNF267 and SGMS2 effect, **F-I** total ceramide and sphingomyelin level detection by ceramide ELISA Kit and sphingomyelin Kit (*N* = 3). **J-M** With expression restoration of ZNF267 or SGMS2 and the use of Ly93 inhibitor, matrigel transwell assays in LARP6-knockdown CRC cells were conducted (**J**-**K**). Statistical results were shown below (**L**-**M**) (*N* = 3). *P < 0.05, **P < 0.01, ***P < 0.001, ns means no statistic difference. The error bars represent Mean ± SD

SGMS2, a key enzyme in sphingomyelin synthesis, transfers phosphocholine from phosphatidylcholine to ceramide to produce sphingomyelin, playing an important role in maintaining cell sphid ngolipid homeostasis, especially between ceramide and sphingomyelin [36–41]. We next examined the influence of LARP6 on ceramide and sphingomyelin level in CRC cells. As shown in Fig. 6A-D, *LARP6* over-expression increased ceramide accumulation but decreased sphingomyelin content in CRC cells, and *LARP6* interference was opposite. Moreover, further studies indicated that the effect of LARP6 on ceramide and sphingomyelin level relies on its expression regulation on ZNF267 and SGMS2 (Fig. 6E-I, S3L-N [see Additional file 2]). Ceramide and sphingomyelin, as bioactive lipids, have important effects on cell survival, apoptosis, autophagy, migration and other processes [36–41]. Therefore, there was a good reason to believe that, ZNF267/SGMS2 axis-mediated sphingomyelin synthesis may play a important role in LARP6 suppression on CRC metastasis. As expected, matrigel transwell assay showed that *LARP6* knockdown promotes CRC cells invasion, but the enhancement is counteracted by expression recovery of ZNF267 and SGMS2, or treatment with the selective sphingomyelin synthase 2 inhibitor Ly93 [42, 43] (Fig. 6J-M, S4A [see Additional file 2]).

### LARP6 upregulates autophagy activity of CRC cells

**Fig. 7:**
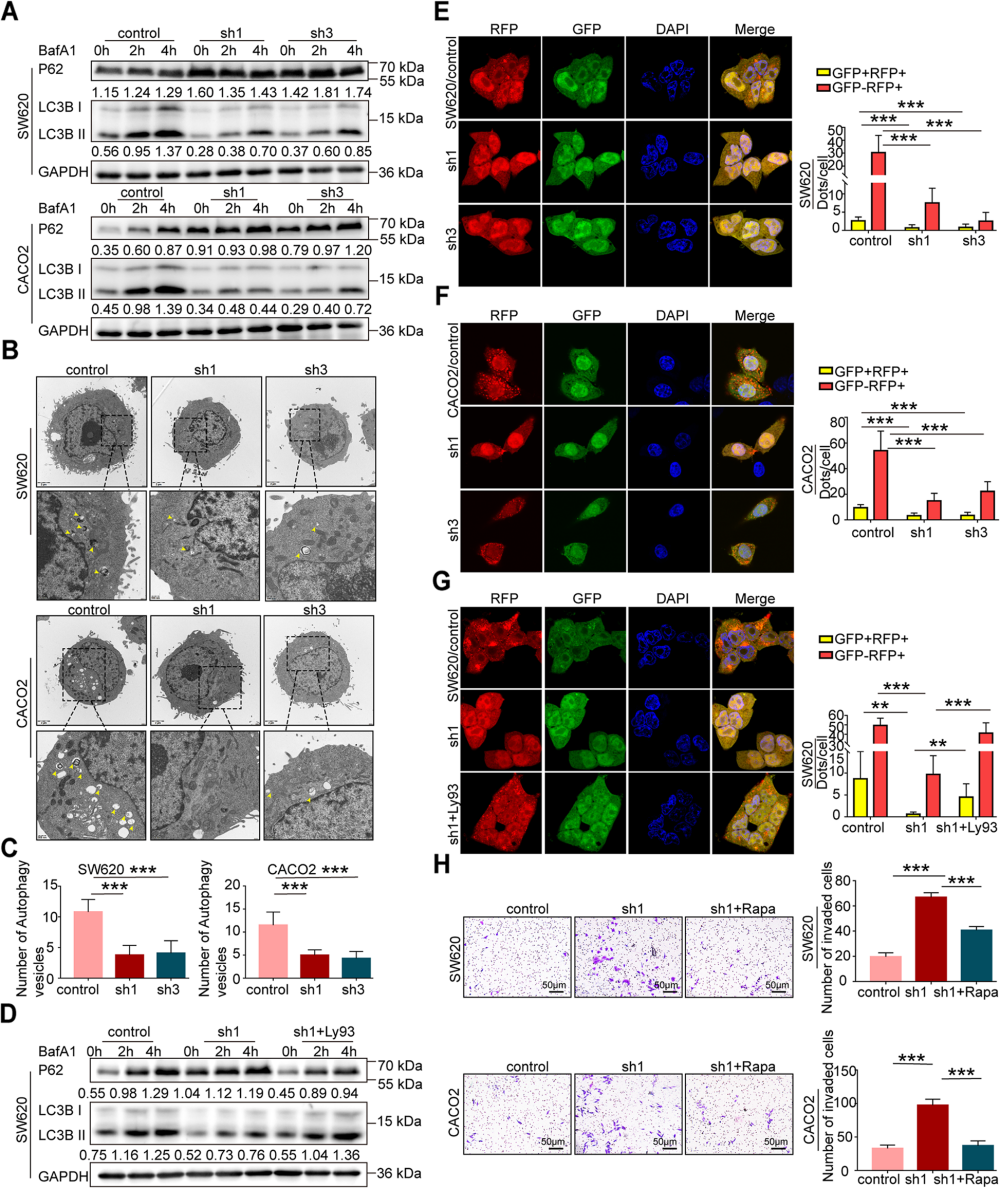
LARP6 upregulates autophagy activity in CRC cells. **A** With or without BafA1, protein expression of LC3B-II and P62 were examined by WB in LARP6-downregulated CRC cells. **B-C** Electron microscopy images presenting the ultrastructure of the CRC cells after LARP6 knockdown. Yellow arrows indicate autophagic vesicles. Statistical results are shown below (*N* = 3). **E-F** After transfected with RFP-GFP-LC3B lentivirus, autophagy flux in CRC cells with LARP6 interference were evaluated using a confocal microscope by quantitation of the number of red and yellow puncta in cells, counting at least 10 cells per group. Red dots indicate autophagolysosomes, and yellow dots represent autophagosomes. Statistical analysis are shown on right pannel (**E**) (*N* = 3). **D** and **G** With the presence of Ly93 inhibitor, protein expression of LC3B-II and P62 were examined (**D**), and the autophagic flux (**G**) was monitored in SW620 cell with stable LARP6 knockdown. Statistical analysis are shown on right pannel (*N* = 3). **H** Matrigel transwell detection in LARP6-knockdown CRC cells with Rapa (100 nM) treatment for 24 h. Statistical results are shown on the right (*N* = 3). *P < 0.05, **P < 0.01, ***P < 0.001, ns means no statistic difference. The error bars represent Mean ± SD

Ceramide is central to sphingolipid metabolism and valid inducer of apoptosis and autophagy [44–47]. Based on the effect of LARP6 on ceramide and sphingomyelin content in CRC cells, we tentatively investigated whether LARP6 influences cell autophagy activity. As shown in Fig. S4B [see Additional file 2], overexpression of *LARP6* increased the abundance of LC3B-II but decreased P62 protein level, and led accumulation of more LC3B-II in the presence of autophagosome and lysosome fusion inhibitor BafA1. Knockdown of *LARP6* showed the opposite results (Fig. 7A). Transmission electron microscopy (TEM) indicated that LARP6 increased the number of autophagic vesicles in CRC cells (Fig. 7B-C, S4D-E [see Additional file 2]). In addition, we transfected a RFP-GFP-LC3 reporter to measure autophagy flux. Abundant red dots (autophagolysosomes) and occasional yellow dots (autophagosomes) were more observed in *LARP6* over-expressed cells (Fig. S4F [see Additional file 2]). However, the number of red dots and yellow dots in *LARP6* knockdown cells were less than that in control group (Fig. 7E-F), which indicating a decrease in autophagy flux. Together, these data all agree that LARP6 upregulates CRC cells autophagy activity. To illustrate the relevance between sphingomyelin synthesis and autophagy, we treated cells with Ly93, a selective sphingomyelin synthetase 2 inhibitor, and found that the inhibitory effect of LARP6 interference on autophagic activity was abolished by this inhibitor (Fig. 7D and G, S4C and S4G [see Additional file 2]). This suggested that SGMS2-mediated sphingomyelin synthesis is essential for LARP6 to regulate autophagy activity in CRC cells.

Related to cell survival, death, migration, metastasis and other numerous cell processes, autophagy is thought to be engaged in cancer development. We also detected the invasion ability of *LARP6* knocked-down cells with the treatment of autophagy agonist Rapa. The results indicated that LARP6 inhibition on CRC metastasis was at least partially depends on the enhancement of autophagy activity (Fig. 7H).

### LARP6 inhibits CRC metastasis through ZNF267/SGMS2 axis

**Fig. 8:**
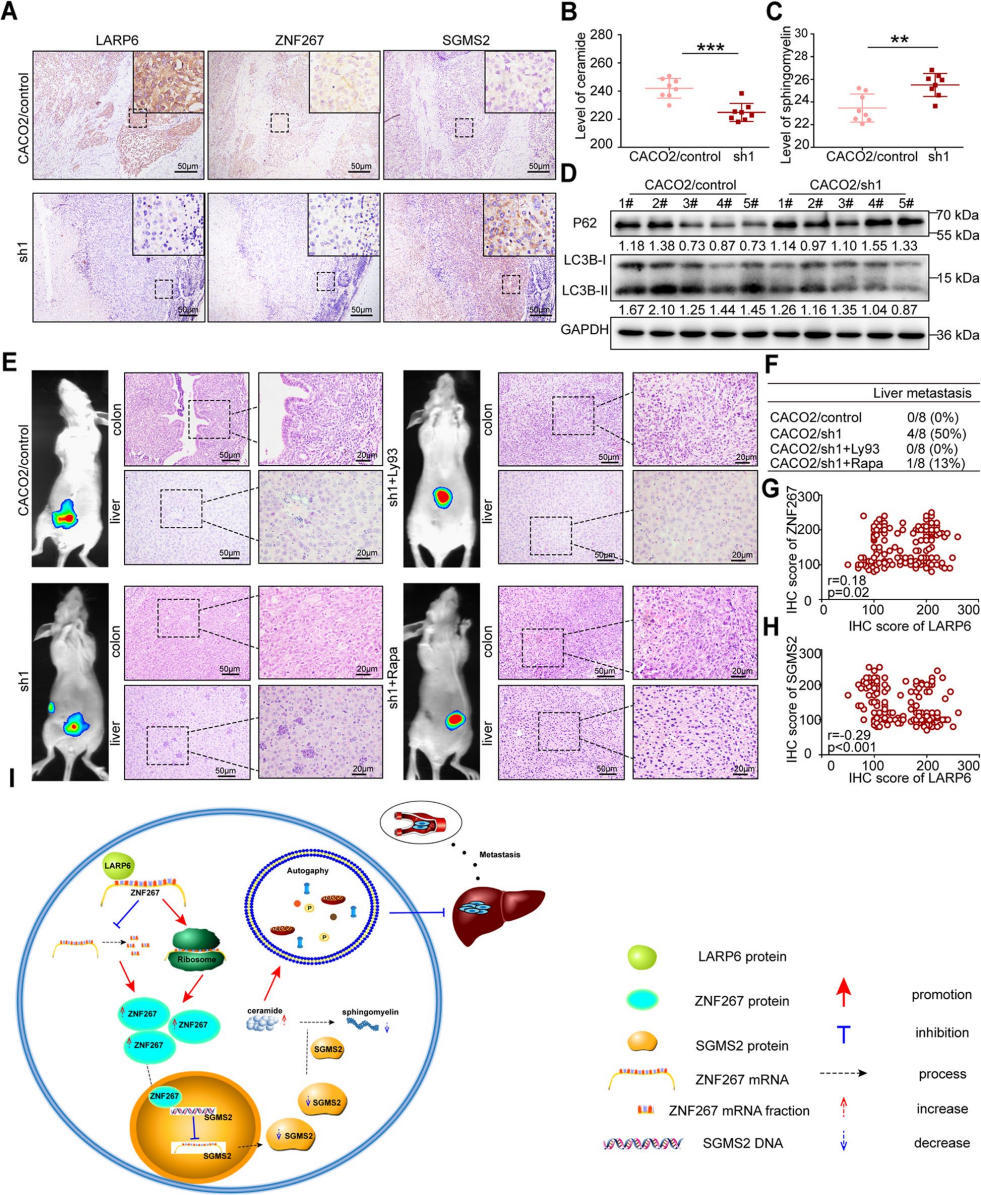
LARP6 suppresses CRC metastasis through ZNF267/SGMS2 axis. **A-D** Analysis of primary CRC tissues in orthotopic CRC mice model constructed with LARP6-interfered CACO2 cells or control cells: IHC staining of LARP6, ZNF267 and SGMS2 (**A**), total ceramide (**B**) and sphingomyelin (**C**) level examination using ceramide ELISA Kit and sphingomyelin Kit, and autophagy detection with LC3B-II and P62 protein (**D**). **E-F** With the use of Ly93 inhibitor and Rapa, in vivo rescue experiment was conducted. (**E**) Representative images of orthotopic CRC mice model: fluorescence in vivo imaging at 60 days after injection (left) and H&E staining of liver tissues and primary colon tumor tissues (right). (**F**) Summary results of the nude mice presenting with liver metastasis between groups of in vivo rescue experiment. **G-H** Correlation analysis of LARP6 with ZNF267 and SGMS2 expression in 165 paraffin-embedded primary CRC tissues and matched adjacent normal tissues. **I** Schematic diagram of our research representing the function and potential mechanism of LARP6 in CRC. *P < 0.05, **P < 0.01, ***P < 0.001, ns means no statistic difference. The error bars represent Mean ± SD

We next validated the molecular mechanisms of LARP6 functions in vivo. Further analysis of primary tumors from orthotopic CRC mice model showed that *LARP6* knockdown group showed a lower LARP6 and ZNF267 expression levels, but higher SGMS2 expression compared with control group (Fig. 8A). Meanwhile, *LARP6* knockdown group also had lower ceramide content and autophagy activity, but higher sphingomyelin content (Fig. 8B-D). In addition, in vivo rescue experiments showed that knockdown of LARP6 promoted metastasis of CRC in vivo, but this effect was reversed by Ly93 and Rapa treatment (Fig. 8E-F).

Besides, we detected ZNF267 and SGMS2 expression in 165 paraffin-embedded primary CRC tissues and matched adjacent normal tissues. As results shown, ZNF267 expression was downregulated in CRC tissues, while SGMS2 was highly expressed (Fig. S4H-I, Table S2–3 [see Additional file 2]). Further correlation analysis of LARP6/ZNF267/SGMS2 expression showed that LARP6 expression is positively correlated with ZNF267 expression but negatively with SGMS2 (Fig. 8G-H). Collectively, our results revealed a LARP6/ZNF267/SGMS2 signaling pathway that inhibited the sphingolipid-related autophagy and CRC metastasis (Fig. 8I).

## Discussion

With numerous reports on RBPs in cancer, RBP’s ability to interact with thousands of RNA makes it a suitable proteome for selective dysregulation in cancer. An interesting hypothesis is that dysregulation of RBP family members may jointly lead to an imbalance in transcriptome of tumor cells, thereby promoting carcinogenicity [23]. Compatible with these findings, in our research, we clarified the suppressive role of LARP6 in CRC metastasis as a RBP.

LARP6, a member of LARP family, is reported to be closely related to the occurrence and development of tumors [2, 15–18]. Here, we found that LARP6 expression is down-regulated in CRC and that LARP6 inhibits CRC cell invasion and metastasis. Transcriptome imbalance is an important way for RBPs to function in tumors [23–28]. Our RIP-seq identified various tumor-related transcripts relevant to LARP6, which further illustrates its importance in CRC progression. ZNF267, a member of the Kruppel like zinc finger family, promotes HCC cell proliferation and migration, but it has not yet been studied in CRC, and little is known about its targeted genes [29–31]. Here, we found that LARP6 binds to *ZNF267* mRNA and promotes its mRNA and protein expression through a mechanism involving mRNA stability and translation regulation, which is partially similar to previous studies on LARP6 [5, 6]. Furthermore, we preliminarily elucidated its inhibitory effect on the migration and invasion of CRC cells. Although the role of *LARP6* and *ZNF267* in CRC differs from that in other tumors, it is frequent in tumor studies that gene distinctively regulates cancer development in different tumor types or even shows opposing effects. Researchers have attributed this functional difference to strong tissue-specific genetic network architecture or context dependency that determines the operational efficacy of gene in different tissues [48, 49]. The highly tissue-specific epigenetic landscape of a given cell type establishes its responsiveness to oncogenic proliferation signals and determines which drivers, somatic copy number changes, and anueploidies are selected during tumorigenesis. This is also true for transcription factors in which the specific epigenetic chromatin landscape in each tissue can directly influence which targets are transcriptionally accessible and thus what outcomes occur.

Furthermore, although our study has updated list of molecules involved in LARP6 post-transcriptional regulation, specific molecular mechanisms by which LARP6 promotes *ZNF267* expression remain to be further explored. Secondly, in addition to *ZNF267*, other molecules in RIP-seq are also worthy of further study, which is crucial to comprehensively elucidate molecular mechanism of LARP6 inhibiting CRC metastasis.

SGMS2 is one of key enzymes involved in sphingomyelin synthesis [36–38], and a study prompts that ZNF267 may target its promoter. We confirmed that ZNF267 indeed binds to *SGMS2* promoter and inhibits its expression, which consists with its predicted transcriptional inhibitory activity [29–31]. Interestingly, LARP6 also has a negative influence on *SGMS2* expression, thus promoting the accumulation of ceramide, and decreasing sphingomyelin content in CRC cells. Although these effects depend on the regulation of *ZNF267* expression, at least we can explain why sphingolipid metabolism exists in KEGG enrichment result of LARP6.

In recent years, studies on relationship between sphingolipid metabolism disorder and cancer development have been emerging one after another, especially on ceramide. A study has reported that ceramide level is reduced in human colon cancer [50]. In addition, deletion of neutral ceramide enzyme (NCDase) increases ceramide level and protects mice from development of colon cancer in carcinogen-induced models [51]. These findings strongly support our results that LARP6 inhibition on CRC metastasis at least partially relies on suppression of SGMS2-mediated sphingomyelin synthesis. More specifically, for the suppression of LARP6/ZNF267/SGMS2 axis on CRC metastasis, direct action of ceramide and sphingomyelin is one reason, and sphingolipid-related autophagy may be another. Although our study links LARP6-mediated post-transcriptional regulation with sphingomyelin synthesis and autophagy, specific details still need to be illustrated in future. Besides, we study suggested that SGMS2-mediated sphingomyelin synthesis may be a potential therapeutic target for CRC patients, specially for those with low LARP6 expression. In vitro and in vivo rescue experiments using Ly93 have initially verified our hypothesis, and more clinical evidence still need to be further studied.

## Conclusion

*LARP6* functions as a suppressor gene in CRC progression. Mechanically, it regulates *ZNF267/SGMS2* axis to induce ceramide and sphingomyelin imbalance and increase autophagy activity in CRC cells. Our study shows how *LARP6/ZNF267/SGMS2* axis influences CRC progression, and contributes to further understanding of the molecular mechanisms underlying CRC development.

## Supplementary Information

**Additional file 1.** Clinical information of all patients in research.

**Additional file 2.** Supplementary figures and tables.

**Additional file 3.** List of peak of RIP-seq and KEGG analysis of peak-related genes.

**Additional file 4.** GO analysis of ZNF267 using TCGA datasets.

## Data Availability

Datasets generated and analyzed during the current study are available from corresponding author on reasonable request.

## Abbreviations

CRC: Colorectal cancer
RBP: RNA-binding protein
GEPIA: Gene expression profiling interactive analysis
H&E: Haematoxylin and eosin
IHC: Immunohistochemistry
KEGG: Kyoto encylopaedia of genes and genomes
LARP6: La ribonucleoprotein domain family member 6
LC3B: Microtubule associated protein 1 light chain 3 beta
qPCR: Quantitative real-time PCR
RIP-seq: High-throughput RNA immunoprecipitation sequencing
SGMS2: Sphingomyelin synthase 2
TCGA: The cancer genome atlas
WB: Western blotting
ZNF267: Zinc finger protein 267
