# Supplementary material for: LARP6 suppresses colorectal cancer progression through ZNF267/SGMS2-mediated imbalance of sphingomyelin synthesis
Source: J Exp Clin Cancer Res. 2023 Jan 24;42:33. doi: 10.1186/s13046-023-02605-4 (PMC9872320; doi:10.1186/s13046-023-02605-4)
Supplement: Supplementary file 2 — Additional file 2. Supplementary figures and tables. [file 13046_2023_2605_MOESM2_ESM.docx]

**Figure S1**


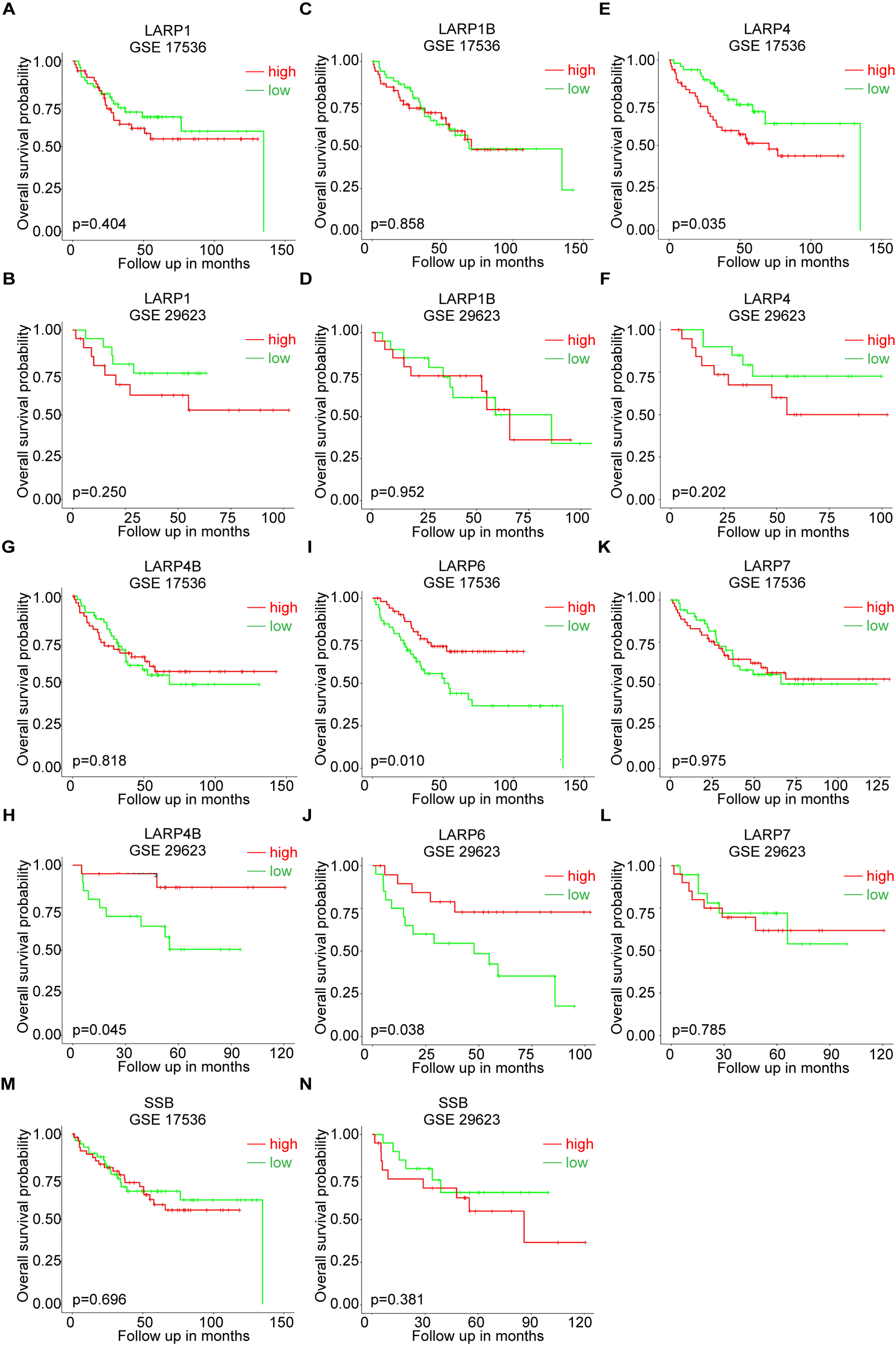


**Figure S1** A-N Overall survival analysis of LARP family members using GEO datasets: LARP1 (A-B), LARP1B (C-D), LARP4 (E-F), LARP4B (G-H), LARP6 (I-J), LARP7 (K-L), SSB (M-N). The upper 30% and the last 30% of the expression level were defined as high expression and low expression respectively. red line indicates high expression and green line indicates low expression.

**Figure S2**


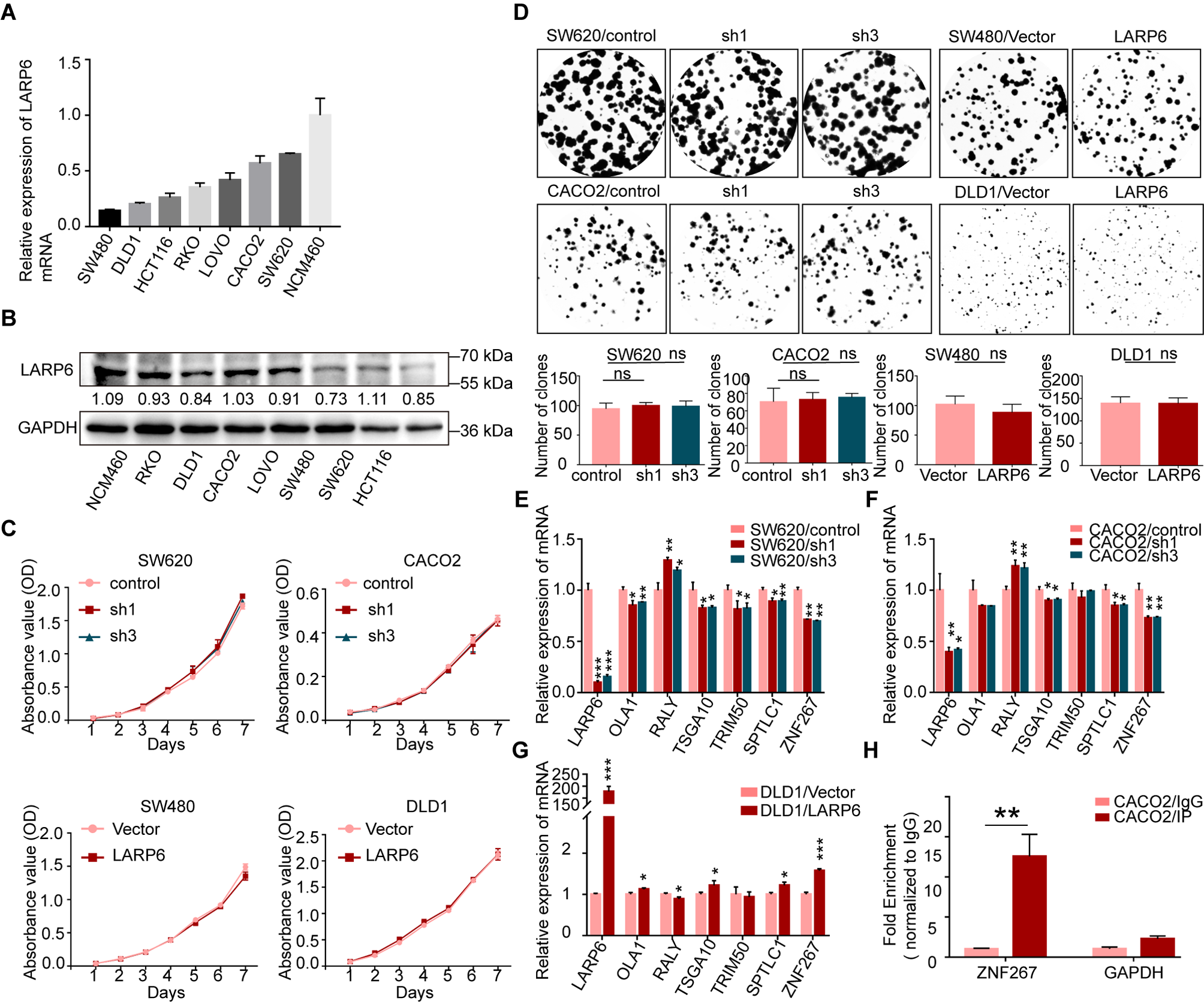


**Figure S2 A-B** LARP6 mRNA (A) and protein (B) expression in 7 CRC cell lines and NCM460 cell. **C** CCK8 assays in LARP6-overexpressed or downregulated CRC cells. **D** Colony formation assays in cells with LARP6 overexpression or interference. Statistical results are shown below (N=3). **E-G** qPCR analysis of candidate genes in CRC cells with LARP6-overexpression (G) or interference (E-F) and control cells. **H** RIP-qPCR assay with anti-LARP6 antibody in CACO2 cells (N=3). GAPDH as a negative control. *P < 0.05, **P < 0.01, ***P < 0.001, ns means no statistic difference. The error bars represent mean ± SD.

**Figure S3**


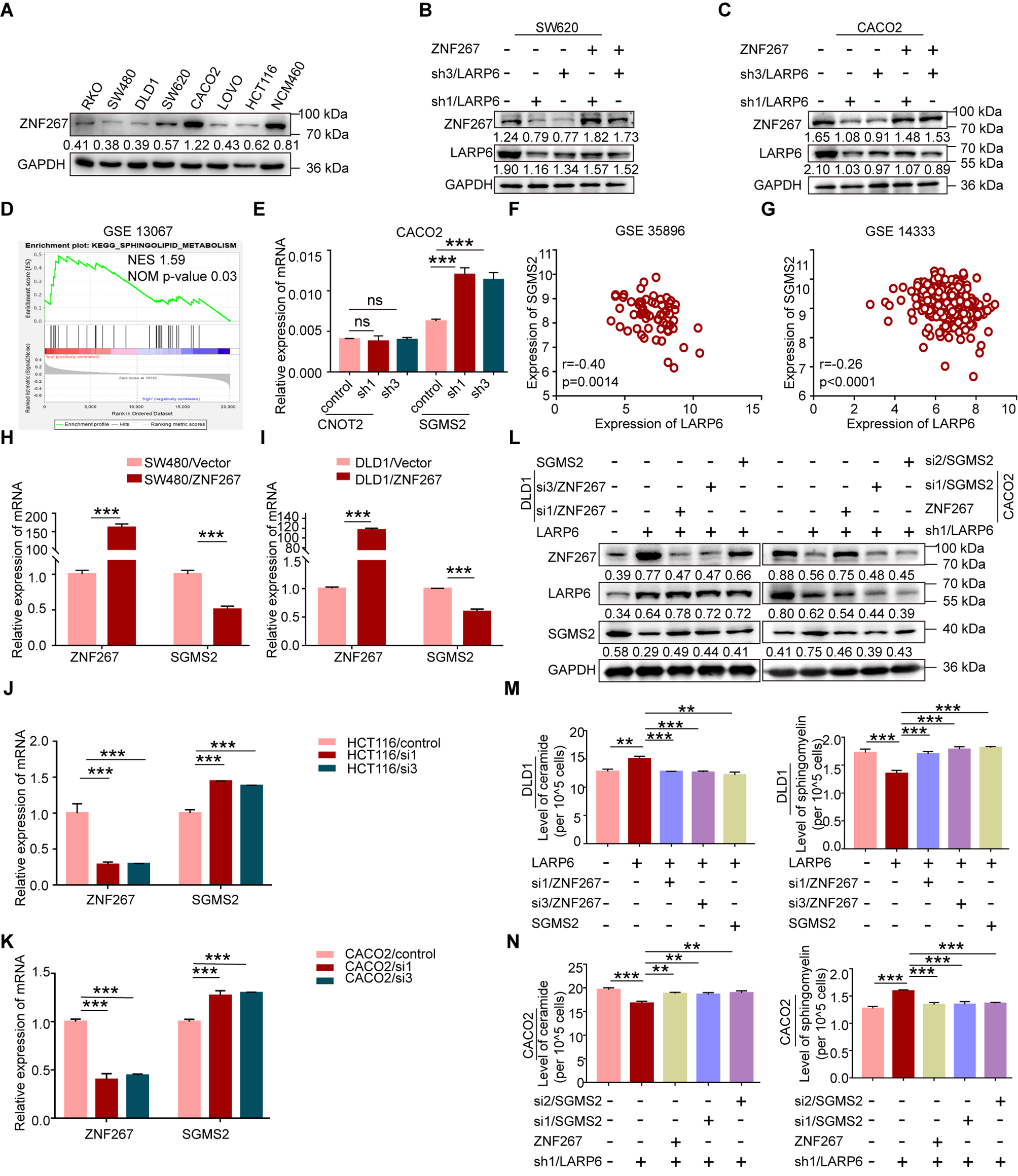


**Figure S3 A** ZNF267 protein expression in 7 CRC cell lines and NCM460 cell. **B-C** Identification of ZNF267 overexpression in SW620 and CACO2 cells with LARP6 knockdown. **D** Relation between LARP6 and sphingolipid metabolism in KEGG enrichment analysis. Sample information were from three different GEO datasets as before. NES: normalized enrichment score. **E** qPCR was used to examine CNOT2 and SGMS2 mRNA expression in CACO2 cells with LARP6 interference. **F-G** Correlation analysis of LARP6 and SGMS2 expression in GEO datasets. r value represents correlation. **H-K** The suppression of ZNF267 on SGMS2 mRNA expression was shown through qPCR. **L-N** In LARP6-overexpressed or interfered CRC cells, total ceramide and sphingomyelin level were detected after forced expression or knockdown of ZNF267 or SGMS2: **L** identification of overexpression or interference of ZNF267 and SGMS2, **M-N** total ceramide and sphingomyelin level detection by ceramide ELISA Kit and sphingomyelin Kit. *P < 0.05, **P < 0.01, ***P < 0.001, ns means no statistic difference. The error bars represent mean ± SD.

**Figure S4**


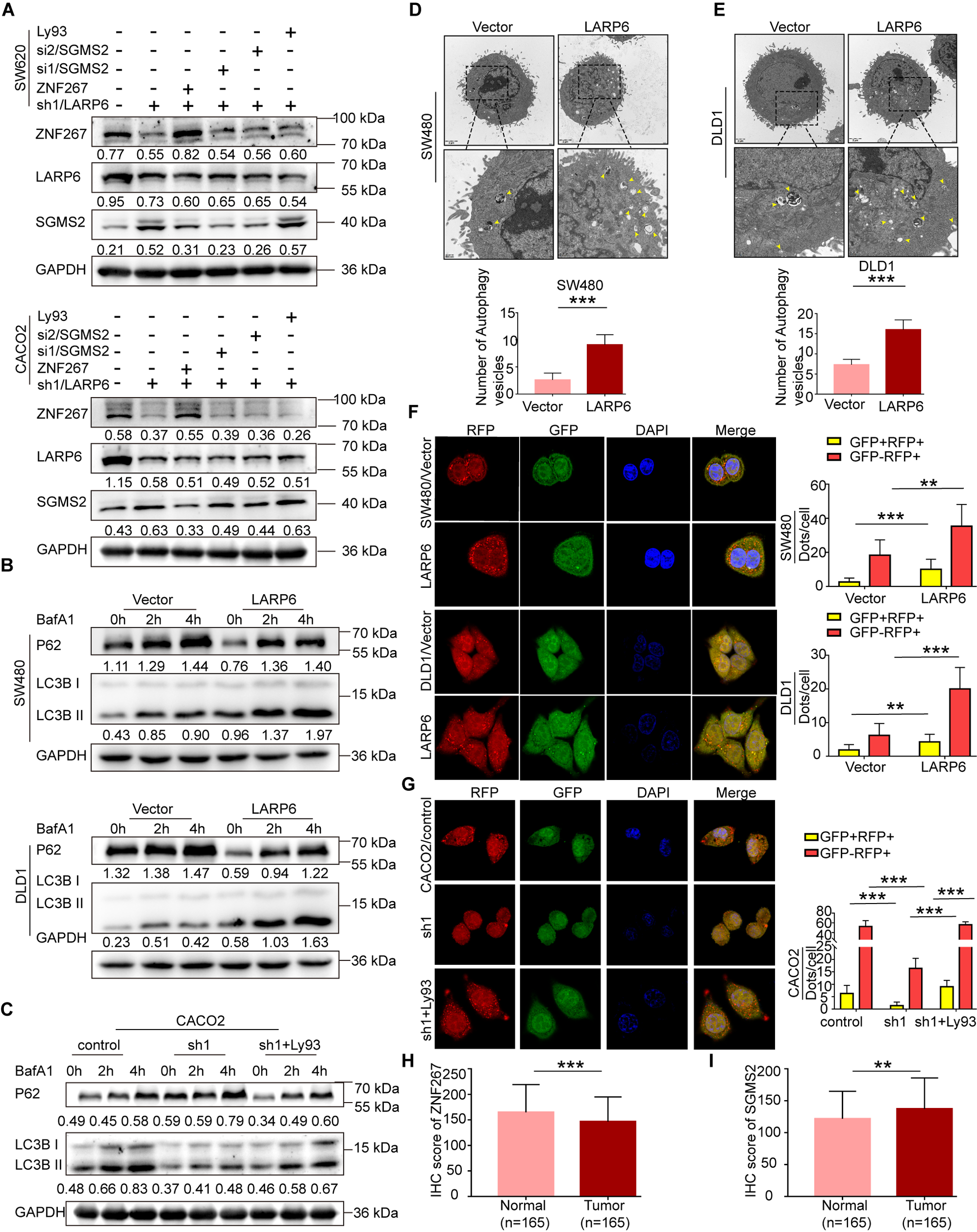


**Figure S4 A** Identification of restoration of ZNF267 or SGMS2 expression in SW620 and CACO2 cells with LARP6 knockdown. **B** With or without BafA1, protein expression of LC3B-II and P62 were examined by WB in LARP6-overexpressed CRC cells. **D-E** Electron microscopy images presenting the ultrastructure of the CRC cells after LARP6 overexpression. Yellow arrows indicate autophagic vesicles. Statistical results are shown below. **F** After transfected with RFP-GFP-LC3B lentivirus, autophagy flux in CRC cells with LARP6 ectopic expression were evaluated using a confocal microscope by quantitation of the number of red and yellow puncta in cells, counting at least 10 cells per group. Red dots indicate autophagolysosomes, and yellow dots represent autophagosomes. Statistical analysis are shown on right pannel. **C and G** With the presence of Ly93 inhibitor, protein expression of LC3B-II and P62 were examined (C) and the autophagic flux (G) was monitored in CACO2 cell with stable LARP6 knockdown. Statistical analysis are shown on right pannel. **H-I** IHC analysis of ZNF267 and SGMS2 in 165 paraffin-embedded primary CRC tissues and matched adjacent normal tissues. *P < 0.05, **P < 0.01, ***P < 0.001, ns means no statistic difference. The error bars represent mean ± SD.

**Table S1 Relationship between LARP6 expression and clinical pathological parameters of CRC**

| **Characteristics** | **n=165(%)** | \| **IHC score of LARP6**  **Mean±SD** \| \| --- \| | **F-value** | **p-value** |
| --- | --- | --- | --- | --- | --- |
| ***Gender*** |  |  | - | **0.382** |
| Male | 92(55.8%) | 160.2±5.6 |  |  |
| Female | 73(44.2%) | 153.2±5.5 |  |  |
|  |  |  |  |  |
| ***Age (years)*** |  |  | - | **0.636** |
| ≤60 | 81(49.1%) | 155.2±5.9 |  |  |
| >60 | 84(50.9%) | 158.9±5.3 |  |  |
|  |  |  |  |  |
| ***Tumor size (cm)*** |  |  | - | **0.570** |
| ≤5 | 103(62.4%) | 158.8±4.7 |  |  |
| >5 | 62(37.6%) | 154.2±7.0 |  |  |
|  |  |  |  |  |
| ***Tumor differentiation*** |  |  | **4.017** | **0.020** |
| High | 12(7.3%) | 190.8±12.2 |  |  |
| Moderate | 128(77.6%) | 157.0±4.5 |  |  |
| Low | 25(15.1%) | 141.4±8.8 |  |  |
|  |  |  |  |  |
| ***T stage*** |  |  | - | **<0.001** |
| T1–2 | 17(10.3%) | 206.5±9.1 |  |  |
| T3–4 | 129(78.2%) | 151.1±4.2 |  |  |
| - | 19(11.5%) |  |  |  |
|  |  |  |  |  |
| ***Lymph node state*** |  |  | - | **<0.001** |
| Negative | 88(53.3%) | 182.6±4.4 |  |  |
| Positive | 77(46.7%) | 128.0±5.0 |  |  |
|  |  |  |  |  |
| ***Distant metastasis*** |  |  | - | **<0.001** |
| Negative | 145(87.9%) | 165.4±3.8 |  |  |
| Positive | 20(12.1%) | 97.0±9.1 |  |  |
|  |  |  |  |  |
| ***Clinical stage*** |  |  | **38.6** | **<0.001** |
| I+II | 83(50.3%) | 183.8±4.3 |  |  |
| III | 62(37.6%) | 138.1±5.4 |  |  |
| IV | 20(12.1%) | 105.3±11.3 |  |  |

**Table S2 Relationship between ZNF267 expression and clinical pathological parameters of CRC**

| **Characteristics** | **n=165(%)** | \| **IHC score of ZNF267**  **Mean±SD** \| \| --- \| | **F-value** | **p-value** |
| --- | --- | --- | --- | --- | --- |
| ***Gender*** |  |  | - | **0.710** |
| Male | 92(55.8%) | 147.8±5.1 |  |  |
| Female | 73(44.2%) | 144.9±5.7 |  |  |
|  |  |  |  |  |
| ***Age (years)*** |  |  | - | **0.118** |
| ≤60 | 81(49.1%) | 140.5±5.3 |  |  |
| >60 | 84(50.9%) | 152.3±5.4 |  |  |
|  |  |  |  |  |
| ***Tumor size (cm)*** |  |  | - | **0.963** |
| ≤5 | 103(62.4%) | 146.7±4.6 |  |  |
| >5 | 62(37.6%) | 146.3±6.7 |  |  |
|  |  |  |  |  |
| ***Tumor differentiation*** |  |  | **1.625** | **0.200** |
| High | 12(7.3%) | 160.0±17.4 |  |  |
| Moderate | 128(77.6%) | 148.0±4.2 |  |  |
| Low | 25(15.1%) | 132.2±9.4 |  |  |
|  |  |  |  |  |
| ***T stage*** |  |  | - | **0.052** |
| T1–2 | 17(10.3%) | 167.1±14.3 |  |  |
| T3–4 | 129(78.2%) | 142.8±4.1 |  |  |
| - | 19(11.5%) |  |  |  |
|  |  |  |  |  |
| ***Lymph node state*** |  |  | - | **0.011** |
| Negative | 88(53.3%) | 155.5±5.2 |  |  |
| Positive | 77(46.7%) | 136.2±5.3 |  |  |
|  |  |  |  |  |
| ***Distant metastasis*** |  |  | - | **<0.001** |
| Negative | 145(87.9%) | 152.7±4.0 |  |  |
| Positive | 20(12.1%) | 102.0±2.4 |  |  |
|  |  |  |  |  |
| ***Clinical stage*** |  |  | **13.660** | **<0.001** |
| I+II | 83(50.3%) | 159.9±5.4 |  |  |
| III | 62(37.6%) | 143.0±5.9 |  |  |
| IV | 20(12.1%) | 101.8±2.5 |  |  |

**Table S3 Relationship between SGMS2 expression and clinical pathological parameters of CRC**

| **Characteristics** | **n=165(%)** | \| **IHC score of SGMS2**  **Mean±SD** \| \| --- \| | **F-value** | **p-value** |
| --- | --- | --- | --- | --- | --- |
| ***Gender*** |  |  | - | **0.216** |
| Male | 92(55.8%) | 141.5±5.1 |  |  |
| Female | 73(44.2%) | 132.1±5.5 |  |  |
|  |  |  |  |  |
| ***Age (years)*** |  |  | - | **0.487** |
| ≤60 | 81(49.1%) | 134.6±5.5 |  |  |
| >60 | 84(50.9%) | 139.9±5.2 |  |  |
|  |  |  |  |  |
| ***Tumor size (cm)*** |  |  | - | **<0.001** |
| ≤5 | 103(62.4%) | 127.7±4.4 |  |  |
| >5 | 62(37.6%) | 153.3±6.4 |  |  |
|  |  |  |  |  |
| ***Tumor differentiation*** |  |  | **3.922** | **0.022** |
| High | 12(7.3%) | 100.4±6.5 |  |  |
| Moderate | 128(77.6%) | 139.8±4.4 |  |  |
| Low | 25(15.1%) | 142.2±9.1 |  |  |
|  |  |  |  |  |
| ***T stage*** |  |  | - | **0.001** |
| T1–2 | 17(10.3%) | 103.5±8.7 |  |  |
| T3–4 | 129(78.2%) | 143.5±4.2 |  |  |
| - | 19(11.5%) |  |  |  |
|  |  |  |  |  |
| ***Lymph node state*** |  |  | - | **0.004** |
| Negative | 88(53.3%) | 127.3±4.7 |  |  |
| Positive | 77(46.7%) | 148.7±5.7 |  |  |
|  |  |  |  |  |
| ***Distant metastasis*** |  |  | - | **<0.001** |
| Negative | 145(87.9%) | 130.1±3.8 |  |  |
| Positive | 20(12.1%) | 189.5±7.9 |  |  |
|  |  |  |  |  |
| ***Clinical stage*** |  |  | **20.37** | **<0.001** |
| I+II | 83(50.3%) | 121.9±4.5 |  |  |
| III | 62(37.6%) | 140.8±6.2 |  |  |
| IV | 20(12.1%) | 190.5±7.7 |  |  |
